# Metformin lowers glucose 6-phosphate in hepatocytes by activation of glycolysis downstream of glucose phosphorylation

**DOI:** 10.1074/jbc.RA120.012533

**Published:** 2020-01-23

**Authors:** Tabassum Moonira, Shruti S. Chachra, Brian E. Ford, Silvia Marin, Ahmed Alshawi, Natasha S. Adam-Primus, Catherine Arden, Ziad H. Al-Oanzi, Marc Foretz, Benoit Viollet, Marta Cascante, Loranne Agius

**Affiliations:** ‡ Biosciences Institute, Newcastle University, Medical School, Newcastle upon Tyne NE2 4HH, United Kingdom; § Department of Biochemistry and Molecular Biomedicine, Faculty of Biology, Universitat de Barcelona, 08007 Barcelona, Spain; ¶ CIBEREHD and Metabolomics Node at INB-Bioinformatics Platform, Instituto de Salud Carlos III, 28029 Madrid, Spain; ‖ INSERM, U1016, Institut Cochin, Paris 75014, France; ** CNRS, UMR8104, Paris 75014, France; ‡‡ Université Paris Descartes, Sorbonne Paris Cité, Paris 75014, France

**Keywords:** liver, metformin, glycolysis, phosphofructokinase, hepatocyte, glucose 6-phosphate

## Abstract

The chronic effects of metformin on liver gluconeogenesis involve repression of the *G6pc* gene, which is regulated by the carbohydrate-response element–binding protein through raised cellular intermediates of glucose metabolism. In this study we determined the candidate mechanisms by which metformin lowers glucose 6-phosphate (G6P) in mouse and rat hepatocytes challenged with high glucose or gluconeogenic precursors. Cell metformin loads in the therapeutic range lowered cell G6P but not ATP and decreased *G6pc* mRNA at high glucose. The G6P lowering by metformin was mimicked by a complex 1 inhibitor (rotenone) and an uncoupler (dinitrophenol) and by overexpression of mGPDH, which lowers glycerol 3-phosphate and G6P and also mimics the *G6pc* repression by metformin. In contrast, direct allosteric activators of AMPK (A-769662, 991, and C-13) had opposite effects from metformin on glycolysis, gluconeogenesis, and cell G6P. The G6P lowering by metformin, which also occurs in hepatocytes from AMPK knockout mice, is best explained by allosteric regulation of phosphofructokinase-1 and/or fructose bisphosphatase-1, as supported by increased metabolism of [3-^3^H]glucose relative to [2-^3^H]glucose; by an increase in the lactate m2/m1 isotopolog ratio from [1,2-^13^C_2_]glucose; by lowering of glycerol 3-phosphate an allosteric inhibitor of phosphofructokinase-1; and by marked G6P elevation by selective inhibition of phosphofructokinase-1; but not by a more reduced cytoplasmic NADH/NAD redox state. We conclude that therapeutically relevant doses of metformin lower G6P in hepatocytes challenged with high glucose by stimulation of glycolysis by an AMP-activated protein kinase–independent mechanism through changes in allosteric effectors of phosphofructokinase-1 and fructose bisphosphatase-1, including AMP, P_i_, and glycerol 3-phosphate.

## Introduction

Metformin is the most commonly prescribed drug for lowering blood glucose in type 2 diabetes. Its therapeutic effect involves inhibition of glucose absorption by the gut and inhibition of glucose production by the liver (1). Evidence for the latter mechanism in man is largely derived from chronic studies demonstrating efficacy after 2–26 weeks therapy (2). Suppression of hepatic glucose production during chronic therapy may involve either acute inhibition of gluconeogenic flux or chronic changes in gene expression (3–5), with various arguments in support of the latter. One such argument is a lack of acute effect of intravenously administered metformin on hepatic glucose production in man (6–8). Studies on metformin effects on gene expression in animal and cellular models have largely focused on mechanisms mediated by activation of AMPK (9). However, repression by metformin of the *G6pc* gene, which encodes the enzyme catalyzing the final reaction in hepatic glucose production, has also been observed in hepatocytes from AMPK-deficient mice (10). The *G6pc* gene is of particular interest because it was identified as a component of the metformin mechanism in both animal diabetes and in man by nontargeted approaches (11–13) and because *G6pc* is regulated by the transcription factor ChREBP (14), which is activated by raised cellular phosphorylated intermediates of glucose metabolism in conditions of raised blood glucose or compromised intracellular homeostasis, resulting in raised glucose 6-phosphate, G6P^4^ (14–17). ChREBP recruitment to the *G6pc* gene promoter is inhibited by metformin in association with lowering of cell G6P and fructose 2,6-P_2_ (18). Although G6P lowering by metformin has been shown in liver *in vivo* (19) and in isolated hepatocytes (18–21), the underlying mechanisms remain unsettled. The aim of this study was to identify the mechanism(s) by which metformin levels corresponding to a therapeutic dose lower G6P in hepatocytes. Such mechanisms are expected to contribute to *G6pc* repression by metformin (10, 18). Various sets of evidence support lowering of G6P by increased glycolysis via allosteric effectors of phosphofructokinase-1.

## Results

### Cell metformin accumulation

**Figure 1.**
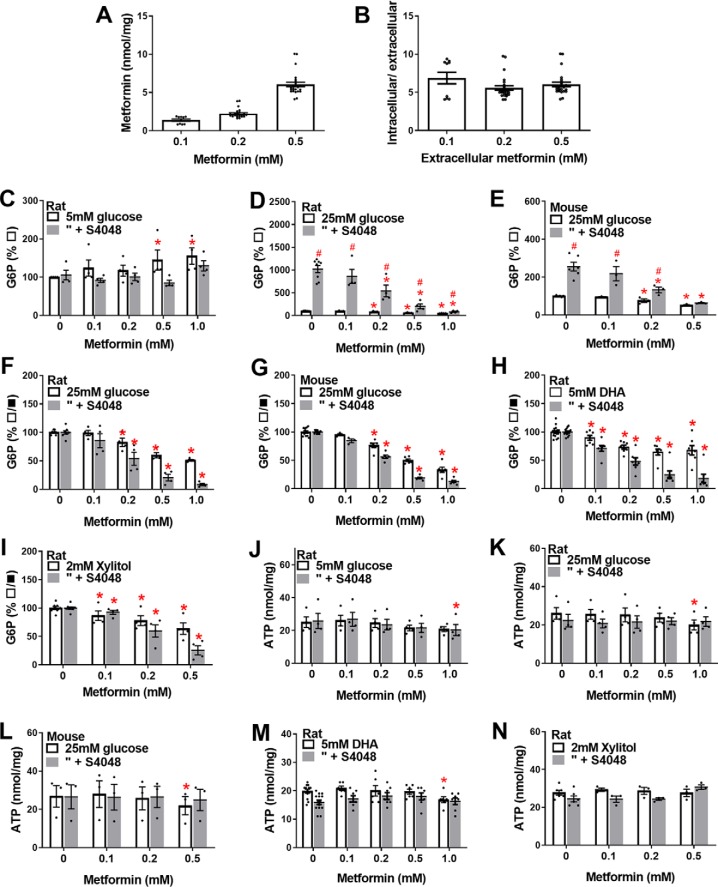
**Metformin accumulation in hepatocytes and effects on cell G6P and ATP.** *A* and *B*, cell metformin in mouse hepatocytes incubated in MEM with 5 mm glucose and [^14^C]metformin at the concentrations indicated for 2 h followed by a further hour with added 25 mm glucose (*n* = 4–9). Cell metformin is expressed as nmol/mg cell protein (*A*) or as intracellular/extracellular concentration ratio (*B*). *C–N*, rat or mouse hepatocytes were incubated for 2 h in MEM containing 5 mm glucose and the metformin concentrations indicated without (*open bars*) or with (*shaded bars*) S4048, followed by a further 1 h with the substrates indicated for determination of G6P (*C–I*) or ATP (*J–M*). Cell G6P is expressed as a percentage of control without S4048 (*C–E*) or a percentage of respective control with or without S4048 (*F–I*). ATP is expressed as nmol/mg protein. *C* and *J*, 5 mm glucose, rat hepatocytes; *D* and *K*, 25 mm glucose, rat hepatocytes; *E* and *L*, 25 mm glucose, mouse hepatocytes; *H* and *M*, 5 mm glucose + 5 mm-DHA; *I* and *N*, 5 mm glucose + 2 mm xylitol; *F* and *G* show data in *D* and *E* normalized to respective control (means ± S.E. for *n* = 3 (*E*, *I*, *L*, and *N*), 4 (*C*, *D*, *J*, and *K*), and 7 (*H* and *M*) hepatocyte preparations). *, *p* < 0.05 effect of metformin (*C–N*); ^#^, *p* < 0.05 effect of S4048 (*C–E*).

Intracellular accumulation of metformin is slower in hepatocytes than in liver *in vivo* (19, 22). Mice given an intragastric load of 50 mg/kg metformin attain a portal vein metformin concentration of 50–60 μm and accumulate peak metformin levels in liver of 1–2 nmol/mg protein within 30 min (22). Rat hepatocytes incubated with 100–200 μm metformin accumulate cell loads of 1–2 nmol/mg protein after 2 h (18). Throughout this study on rat and mouse hepatocytes, we used a protocol comprising a 2-h preincubation with metformin followed by a 1-h incubation with medium containing the substrates and the same metformin concentration as during the preincubation. Using this protocol, the cell metformin content at the end of the 3-h incubation with 100–200 μm metformin is 1–2 nmol/mg in mouse hepatocytes (Fig. 1*A*). This corresponds to an intracellular/extracellular metformin concentration ratio of ∼5 (Fig. 1*B*). These data on mouse hepatocytes (Fig. 1, *A* and *B*) are similar to data on rat hepatocytes reported previously (18).

### Metformin lowers cell G6P when raised with high glucose or gluconeogenic precursors

We determined the effects of metformin on cell G6P in rat or mouse hepatocytes incubated with either high glucose (25 mm) or with dihydroxyacetone (DHA), which enters the glycolytic/gluconeogenic pathway at the level of triose phosphates, or with xylitol, which enters the pathway at triose phosphate and fructose 6-phosphate (23). These experiments were performed without or with the chlorogenic acid derivative S4048 (24), which inhibits the G6P transporter (*Slc37a4*) on the endoplasmic reticulum. S4048 has no effect of G6P levels in hepatocytes incubated with 5 mm glucose (Fig. 1*C*), but it enhanced the elevation by high glucose (25 mm) in rat hepatocytes (Fig. 1*D*) and mouse hepatocytes (Fig. 1*E*). The elevation in G6P by S4048 in hepatocytes and *in vivo* (24–26) supports the role of glucose 6-phosphatase in maintaining G6P homeostasis (16, 17). Metformin did not lower G6P in hepatocytes incubated with 5 mm glucose (Fig. 1*C*), but it lowered G6P dose-dependently in rat or mouse hepatocytes with 25 mm glucose (Fig. 1, *D* and *E*) and caused a greater fractional lowering when G6P was elevated by S4048 (Fig. 1, *F* and *G*). Similarly metformin lowered G6P with both DHA (Fig. 1*H*) and xylitol (Fig. 1*I*). Cell ATP was decreased by 1 mm metformin but not by ≤0.5 mm metformin (Fig. 1, *J–N*).

### AMPK activators do not mimic metformin on glycolysis, gluconeogenesis, or cell G6P

**Figure 2.**
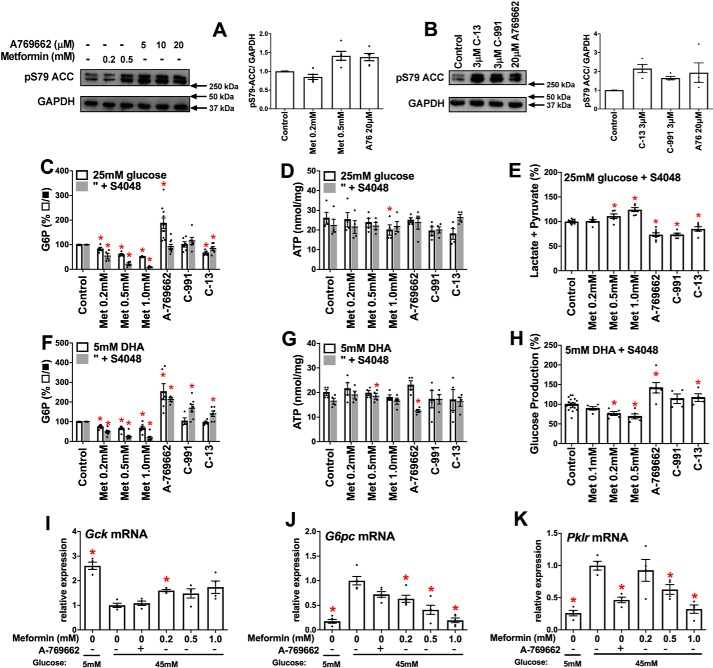
**Opposite effects of AMPK activators and metformin on glycolysis and gluconeogenesis in rat hepatocytes.** Incubations with metformin or AMPK activators were for 2 h followed by 1 h of incubation with substrate as in Fig. 1. *A* and *B*, phosphorylation of *ACC*, acetyl-CoA carboxylase–S79 by metformin and A-769662 (*A*) and by the three AMPK activators: C13, C-991, and A-769662 (*B*). Representative blots and densitometry are shown. *C–E*, cell G6P and ATP and production of lactate + pyruvate with 25 mm glucose. *F* and *G*, cell G6P and ATP with 5 mm DHA and 5 mm glucose. *H*, glucose production from 5 mm DHA in glucose-free medium. *I–K*, *Gck*, *G6pc*, and *Pklr* mRNA in rat hepatocytes after 4 h of incubation with the additions indicated at 5 or 45 mm glucose. The values are the means ± S.E. for *n* = 4–6 (*A* and *B*), 4–10 (*C–H*), and 4–5 (*I–K*). *, *p* < 0.05 relative to respective control (*C–H*) or relative to high glucose control (*I–K*).

We next tested whether direct activators of AMPK (A-769662, 991, and C-13) mimic the metformin lowering of G6P with high glucose or DHA (Fig. 2). A-769662 and compound 991 bind to a discrete pocket between the α and β subunits of AMPK (27, 28), whereas C-13 is a prodrug that is metabolized to an “AMP mimetic” that binds to the γ-regulatory subunit (29). A-769662 (20 μm) caused similar phosphorylation of the AMPK target acetyl-CoA carboxylase–S79 as 500 μm metformin (Fig. 2*A*), and C-13 and 991 at 3 μm caused comparable phosphorylation as 20 μm A-769662 (Fig. 2*B*). With 25 mm glucose, A-769662 increased G6P, 991 had no effect, and C-13 caused a modest decrease compared with metformin (Fig. 2*C*) with negligible effect on ATP (Fig. 2*D*) but with significant inhibition in the production of lactate and pyruvate unlike metformin (Fig. 2*E*). With DHA, the AMPK activators unlike metformin significantly raised G6P (Fig. 2*F*), with little effect on ATP (Fig. 2*G*), and A-769662 and C-13 also increased glucose production (Fig. 2*H*). This indicates opposite effects of AMPK activators from metformin on glycolysis with high glucose and on gluconeogenesis from DHA and shows that the AMPK activators do not mimic the G6P lowering by metformin.

### A-769662 does not mimic low metformin on G6pc and Gck expression at high glucose

We next tested whether A-769662 mimics metformin (≥0.2 mm) on gene regulation at high glucose. For these experiments, we used 45 mm glucose to achieve G6P levels intermediate between 25 mm glucose alone and 25 mm glucose + S4048. High glucose caused repression of *Gck* (by 60%) and induction of *G6pc* and *Pklr* by 5- and 3-fold, respectively (Fig. 2, *I–K*). A-769662 caused similar *Pklr* repression as high metformin (Fig. 2*K*) but did not mimic the effect of 0.2 mm metformin on either *Gck* or *G6pc* expression (Fig. 2, *I* and *J*). This implicates AMPK-independent mechanisms for the counter-regulatory effects of low metformin on *G6pc* and *Gck* expression.

### Metformin lowers G6P in hepatocytes from AMPK-KO mice

**Figure 3.**
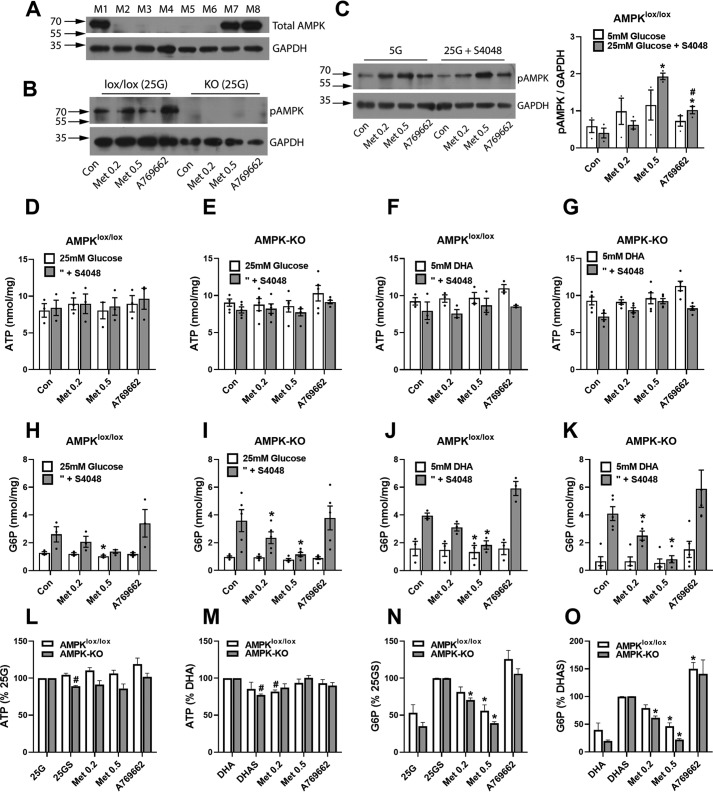
**Metformin lowers G6P in hepatocytes from AMPK-KO mice.** *A*, immunoactivity to AMPK in hepatocytes from AMPKα1α2^lox/lox^ mice (M1, M7, and M8) and AMPKα1α2^lox/lox^ Alb-CRE (M2–M6) mice designated AMPK-KO. *B*, immunoactivity to AMPK-T172(P) after 3 h of incubation with metformin (0.2 or 0.5 mm) or A-769662 (10 μm) in hepatocytes from AMPKα1α2^lox/lox^ control and AMPK-KO mice. *C*, immunoactivity to AMPK-T172(P) in hepatocytes from AMPKα1α2^lox/lox^ incubated for 3 h with or without metformin (0.2 or 0.5 mm) or A-769667 (10 μm) at either 5 or 25 mm glucose + S4048, representative immunoblot and densitometry for *n* = 3 mice. *, *p* < 0.05 *versus* respective control; ^#^, *versus* respective 5 mm glucose. *D–O*, hepatocytes from AMPK^lox/lox^ (*n* = 3) or AMPK-KO (*n* = 5) mice were preincubated for 2 h with or without metformin (0.2 or 0.5 mm) or A-769662 (10 μm) for 2 h followed by 1 h of incubation in medium with either 25 mm glucose with or without S4048 or with 5 mm DHA with or without S4048 for determination of cell ATP (*D–G*) and G6P (*H–K*) expressed as nmol/mg protein. *L* and *M*, ATP from treatments with 25 mm glucose + S4048 expressed as a percentage of controls without S4048. *N* and *O*, G6P from treatments with 25 mm glucose + S4048 expressed as percentages of control with S4048. *, *p* < 0.05 *versus* respective control; ^#^, *versus* substrate control without S4048 (*S*). *Con*, control.

To test for involvement of AMPK in the metformin mechanism on G6P, we used hepatocytes from liver-specific AMPKα1α2 knockout mice. We confirmed the lack of immunoactivity to AMPKα in hepatocytes from AMPKα1^lox/lox^,α2^lox/lox^–Alfp–Cre (AMPK-KO) compared with the AMPKα1^lox/lox^,α2^lox/lox^ (AMPK^lox/lox^) controls (Fig. 3*A*) and also a lack of immunoactivity to phospho-AMPKα after challenge with metformin or A-769662 (Fig. 3*B*). To test whether activation of AMPK by metformin is affected by raised G6P, we compared phospho-AMPK immunoactivity in conditions of low and high G6P with 25 mm glucose + S4048 in control hepatocytes from AMPK^lox/lox^ mice. Phosphorylation of AMPK by high metformin (0.5 mm) and by A-769662 (10 μm) was not attenuated by high glucose + S4048 compared with 5 mm glucose (Fig. 3*C*). Hepatocytes from AMPK-KO mice had similar levels of cell ATP in control conditions without S4048 with either 25 mm glucose (Fig. 3, *D* and *E*) or with 5 mm DHA (Fig. 3, *F* and *G*) and similar elevation of G6P with S4048 at 25 mm glucose or DHA (Fig. 3, *H–K*) as control hepatocytes from AMPK^lox/lox^ mice. The AMPK-KO hepatocytes showed small but significant lowering of ATP with S4048 (Fig. 3, *L* and *M*), which is best explained by compromised ATP homeostasis in conditions of raised G6P. Metformin (0.2 and 0.5 mm) caused similar or greater fractional lowering of G6P in AMPK-KO hepatocytes with high glucose + S4048 or with DHA + S4048 as for AMPK^lox/lox^ controls (Fig. 3, *N* and *O*). This indicates involvement of AMPK-independent mechanisms in the metformin lowering of G6P.

### Rotenone, an uncoupler, and an NNT inhibitor lower G6P

**Figure 4.**
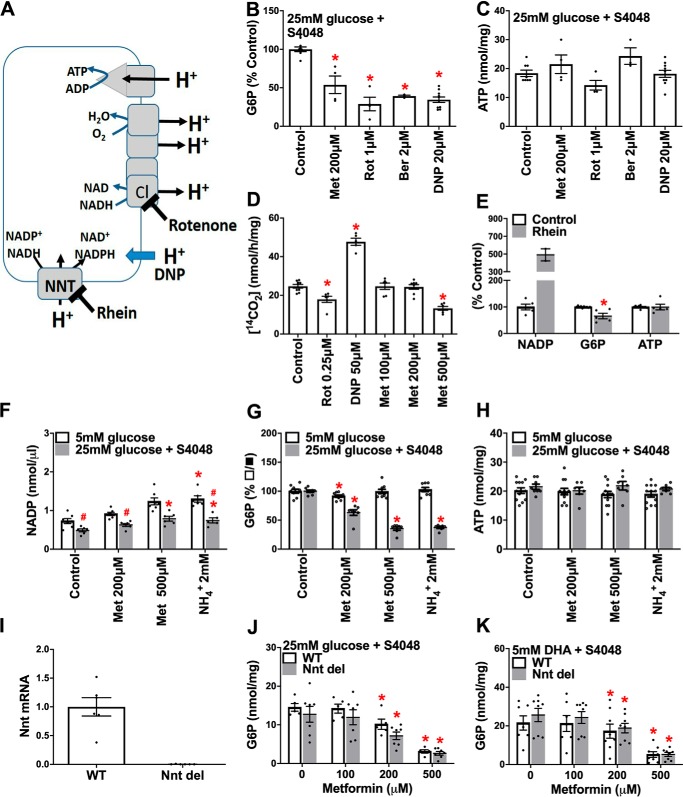
**Rotenone, dinitrophenol, and rhein mimic the G6P lowering by metformin.** *A*, target sites of mitochondrial inhibitors: rotenone, complex 1 (*C1*); DNP, uncoupler (dissipation of proton gradient); and rhein, inhibitor of NNT. *B–K*, incubations with metformin and mitochondrial inhibitors were for 2 h followed by 1 h of incubation with substrate as in Fig. 1. *B* and *C*, cell G6P and ATP in rat hepatocytes incubated with mitochondrial inhibitors. The values are means ± S.E. for *n* = 3–15. *D*, glucose oxidation in mouse hepatocytes incubated with 15 mm [U-^14^C]glucose for 1 h (after 2 h with or without metformin). The values are means ± S.E. for *n* = 3. *E*, rhein raises cell NADP and lowers G6P (*n* = 5 G6P,ATP; 2 NADP). *F–H*, effects of metformin and 2 mm NH_4_Cl on NADP, G6P, and ATP (*n* = 4). *I–K*, metformin lowers G6P in hepatocytes from mice with either an intact (WT) or lacking a functional Nnt gene (*Nnt del*; *n* = 7–8; *J* and *K*). The values are means ± S.E. *, *p* < 0.05 *versus* respective control (*B–K*); ^#^, *p* < 0.05, 25 mm *versus 5 m*m glucose (*F*).

To test for mechanisms linked to mitochondrial function (Fig. 4*A*), we compared metformin with a complex 1 inhibitor (rotenone), an uncoupler (dinitrophenol (DNP)), and with berberine, which causes mitochondrial depolarization similar to metformin (30). Rotenone, DNP, and berberine caused comparable lowering of G6P as metformin (Fig. 4*B*), with negligible effect on ATP (Fig. 4*C*). To test whether the metformin mechanism is similar to that of rotenone or the uncoupler, we determined the metabolism of [U-^14^C]glucose to ^14^CO_2_ as a measure of mitochondrial oxidation (Fig. 4*D*). Glucose oxidation was inhibited by rotenone, as expected for a complex I inhibitor, which promotes an increase in NADH/NAD ratio, and it was stimulated with DNP, consistent with dissipation of the proton gradient and increased electron transport. Metformin inhibited glucose oxidation at 500 μm (Fig. 4*D*), consistent with inhibition of complex I at this concentration (31). However, lower metformin concentrations had no effect on glucose oxidation (Fig. 4*D*). The similar lowering of G6P by rotenone and DNP, which have opposite effects on substrate oxidation, implicates the decrease in the mitochondrial proton gradient as a possible link to the G6P lowering. Mechanisms linked to the mitochondrial proton gradient include ATP synthase (complex V) and nicotinamide nucleotide transhydrogenase (NNT), which generates NADPH from NADH, NADP, and proton import (Fig. 4*A*). We next tested whether rhein (4,5-dihydroxyanthraquinone 2-carboxylic acid), an inhibitor of NNT (32), lowers G6P. Rhein (40 μm) raised NADP, as expected (33), and lowered G6P in conditions of maintained ATP (Fig. 4*E*), suggesting a possible role for either NNT inhibition or for the raised NADP/NADPH ratio.

### Metformin raises NADP but NNT deletion does not abolish the G6P-lowering effect of metformin

To test for possible involvement of NNT activity or raised NADP in the G6P-lowering effect of metformin, we determined cell NADP in incubations with metformin, and we also tested NH_4_^+^ (2 mm), which raises NADP by consumption of NADPH during urea synthesis (33, 34). Cell NADP was higher at 5 mm glucose than at 25 mm glucose and was raised by NH_4_^+^ at low and high glucose and by 500 μm metformin at high glucose (Fig. 4*F*). NH_4_^+^, like metformin, lowered G6P at high glucose (Fig. 3*G*) with no effect on ATP (Fig. 4*H*). The raised NADP by metformin and the lowering of G6P by rhein and NH_4_^+^, which raise NADP by different mechanisms (33, 34), support potential roles for compromised NNT activity or for the raised NADP/NADPH in the G6P lowering, for example by increased activity of the pentose pathway, which uses G6P and NADP as substrates. To test for a role of NNT we determined the effects of metformin in hepatocytes from mice with a deletion in the *Nnt* gene (35) (Fig. 4*I*). The lowering of G6P by metformin was similar in hepatocytes without or with a functional *Nnt* gene (Fig. 4, *J* and *K*). This indicates involvement of mechanisms other than NNT inhibition in the G6P depletion by metformin.

### Changes in glucose phosphorylation or glycogen metabolism cannot explain the metformin lowering of G6P

**Figure 5.**
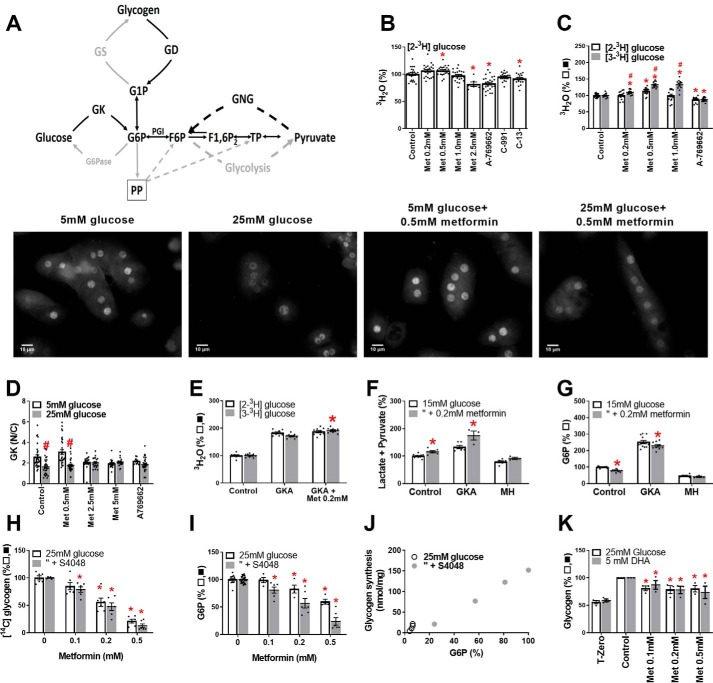
**G6P lowering by metformin is not explained by inhibition of glucose phosphorylation or stimulation of glycogen storage.** *A*, lowering of G6P can occur by inhibition of G6P producing or stimulation of G6P-consuming pathways. *B–K*, incubations with metformin and activators or inhibitors were for 2 h followed by 1 h of incubation with substrate and radiolabel. *B*, metabolism of [2-^3^H]glucose in rat hepatocytes (*n* = 7), except 2.5 mm metformin (*n* = 2). *C*, metabolism of [2-^3^H]glucose and [3-^3^H]glucose in mouse hepatocytes (*n* = 4–5). *D*, glucokinase translocation 25 mm *versus* 5 mm glucose (*n* = 20–30 fields in 2–3 rat hepatocyte preparations) and representative images above. *Scale bars*, 10 μm. *E–G*, mouse hepatocytes: effects of metformin (0.2 mm) in combination with a GKA (10 μm Ro-20-1675) or mannoheptulose (*MH*, 10 mm) on metabolism of [2-^3^H],[3-^3^H]glucose (*E*), lactate and pyruvate (*F*), and cell G6P (*G*) (*n* = 3–9). *H–J*, glycogen synthesis and cell G6P: concentration-dependent inhibition of glycogen synthesis by metformin and correlation with G6P (*n* = 3). *K*, metformin inhibits glycogen storage with high glucose or DHA (*n* = 3). *, *p* < 0.05 effect of metformin or AMPK activator; ^#^, *p* < 0.05 [3-^3^H]glucose *versus* [2-^3^H]glucose (*C*) or 25 mm glucose *versus* 5 mm glucose (*D*).

We tested which candidate metabolic pathways are involved in the metformin mechanism (Fig. 5*A*). Depletion of G6P may result from inhibition of G6P generating pathways (*e.g.* glucose phosphorylation, gluconeogenesis, or glycogenolysis) or stimulation of G6P-consuming pathways (glycogen synthesis, glycolysis, or pentose pathway). We used [2-^3^H]glucose to measure glucose phosphorylation and [3-^3^H]glucose to determine G6P metabolism by glycolysis and the pentose pathway. Glucose phosphorylation was modestly inhibited by A-769662 and C-13 and by 2.5 mm metformin but not by lower metformin (0.2–1 mm) (Fig. 5, *B* and *C*). Similarly glucokinase translocation by high glucose was inhibited by 2.5–5 mm metformin but not by 0.5 mm metformin (Fig. 5*D*). Detritiation of [3-^3^H]glucose (Fig. 5*C*, *shaded bars*) relative to [2-^3^H]glucose was increased by metformin (0.2–1 mm), indicating stimulation of glycolysis and/or pentose pathway after G6P formation.

In the presence of a glucokinase activator (GKA) to maximally activate endogenous glucokinase, low metformin also modestly increased [3-^3^H]glucose metabolism (Fig. 5*E*) and formation of lactate and pyruvate (Fig. 5*F*), whereas in the presence of mannoheptulose, a glucokinase inhibitor, the effects of metformin on pyruvate and lactate formation (Fig. 5*F*) and on G6P (Fig. 5*G*) were abolished. Cumulatively, this implicates metformin stimulation of G6P disposal by glycolysis and/or the pentose pathway downstream of glucose phosphorylation at endogenous or raised but not attenuated glucokinase activity.

Lowering of G6P can occur in conditions of stimulation of glycogen synthesis (36). We therefore tested the effects of metformin on glycogen synthesis (Fig. 5*H*). Metformin caused concentration-dependent inhibition of glycogen synthesis (Fig. 5*H*), and this correlated with the G6P lowering (*r* = 0.99; Fig. 5, *I* and *J*). Metformin also inhibited glycogen storage with 25 mm glucose and DHA as substrates (Fig. 5*K*). G6P is a major regulator of glycogen synthesis (37). These results rule out a role for changes in glycogen metabolism as a cause of G6P depletion but implicate the G6P lowering as the cause of the impaired glycogen synthesis.

### Flux through glycolysis and the pentose pathway determined with [1,2-^13^C_2_]glucose: converse effects of metformin and AMPK activators

To test whether metformin increases flux through the pentose pathway, we first used [1-^14^C]glucose and [6-^14^C]glucose to estimate flux from the difference in ^14^CO_2_ formation ([1-^14^C] minus [6-^14^C]glucose). However, decarboxylation of [1-^14^C]glucose was lower than from [6-^14^C]glucose. This was not due to impurities in the [1-^14^C]glucose but to lack of equilibration at the triose phosphate isomerase step (38). This was confirmed from incubation with ethanol (39), which inhibited ^14^CO_2_ formation from [6-^14^C]glucose but not [1-^14^C]glucose (results not shown).

**Figure 6.**
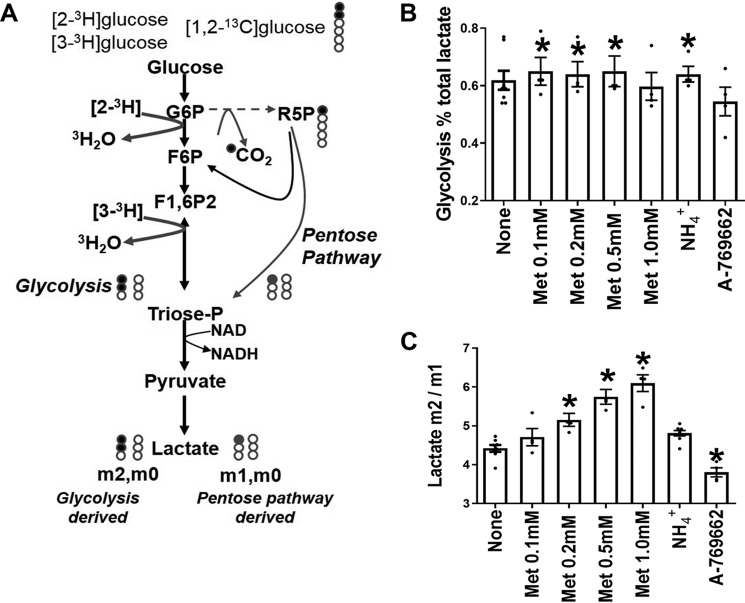
**Metformin but not AMPK activators stimulates glycolysis.** *A*, metabolism of [1,2-^13^C_2_]glucose by glycolysis (to m2, m0 lactate) and pentose pathway (m1, m0 lactate). *B* and *C*, rat hepatocytes were incubated for 1 h in MEM with 2 μm S4048, 0.4 mm α-cyanocinnamate, 0.5 mm AOA, and [1,2-^13^C_2_]glucose (15 mm) for determination of lactate m0, m1, and m2 mass isotopologs. Metformin and A-769662 were present during the final 1 h of incubation and during a 2-h preincubation. NH_4_Cl (2 mm) was present during the final 1 h of incubation. The values are means ± S.E. for *n* = 4 hepatocyte preparations. *, *p* < 0.05 relative to control.

We next used [1,2-^13^C_2_]glucose to measure partitioning of flux between glycolysis (via PFK1) and the pentose pathway (via glucose 6-phosphate dehydrogenase) from [^13^C]lactate mass isotopologs: m0, m1, and m2, where m0 represents unlabeled lactate, and m1 and m2 represent lactate with one or two ^13^C atoms, respectively (Fig. 6*A*). Metabolism of [1,2-^13^C_2_]glucose by the pentose pathway generates m1 and m0 lactate, whereas glycolysis generates m2 and m0 lactate. Pyruvate cycling in mitochondria would lead to conversion of m2 to m1. Incubations were performed with α-cyano-4-hydroxycinnamate and amino-oxyacetate to inhibit substrate entry into mitochondria as pyruvate or alanine (40) in medium without glutamine. We confirmed inhibition of entry of glucose carbon into mitochondria from the lack of ^13^C incorporation into glutamate. The medium also contained S4048 to prevent flux through glucose 6-phosphatase. Comparison of glucose isotopologs at the start and end of the incubation confirmed no change in ^13^C distribution in medium glucose. In these conditions the ratio of m2/m1 lactate is a measure of the relative G6P flux by glycolysis (PFK1) *versus* pentose pathway (Fig. 6*A*). Metformin (100–500 μm) had two effects on [^13^C]lactate mass isotopolog distribution. It increased the fraction of lactate derived from glucose (Fig. 6*B*), and it increased the m2/m1 ratio (Fig. 6*C*). The AMPK activator decreased the m2/m1 ratio (Fig. 6*C*). Cumulatively metformin stimulates glycolysis via PFK1 in absolute terms and relative to flux via the pentose pathway, whereas the AMPK activator had the converse effect on the m2/m1 ratio. NH_4_^+^ (2 mm) increased the fraction of lactate derived from glucose without changing the m2/m1 ratio, indicating increased glycolysis and possibly also pentose pathway.

### Modulation of cell G6P by inhibition of PFK-1 or FBP1 activity

**Figure 7.**
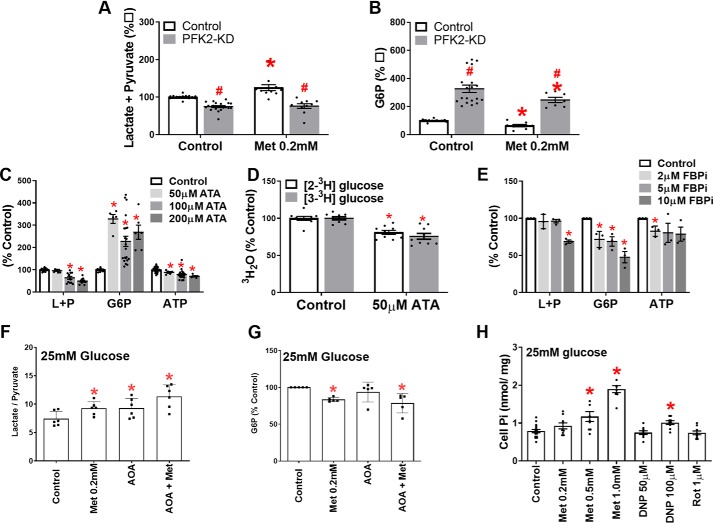
**Changes in hepatocyte G6P during selective targeting of PFK1 and/or FBP1.** *A* and *B*, mouse hepatocytes were either untreated or treated with an adenoviral vector (PFK-KD) to deplete fructose 2,6-P_2_ and after overnight culture were incubated for 2 h with S4048 and without or with 0.2 mm metformin and for a further 1 h with 25 mm glucose. *A*, lactate + pyruvate production. *B*, cell G6P (*n* = 7–10). *C* and *D*, mouse hepatocytes were incubated with S4048 and the concentrations of ATA indicated for 2 h and then for 1 h with 25 mm glucose. *C*, cell G6P and ATP and lactate + pyruvate. *D*, metabolism of [2-^3^H]glucose and [3-^3^H]glucose. *E*, incubations were as for *C* but with the FBP1 inhibitor (FBPi) concentrations as indicated. *F* and *G*, mouse hepatocytes were incubated for 2 h without or with 200 μm metformin and then for a further 1 h with 25 mm glucose without or with 100 μm AOA (*n* = 5–6). *H*, cell P_i_ in mouse hepatocytes (*n* = 3) after 2 h of incubation with metformin and inhibitors and for a further 1 h with 25 mm glucose.*, *p* < 0.05 effect of metformin or inhibitor; ^#^, *p* < 0.05 effect of PFK-KD.

To identify candidate mechanisms for the G6P lowering, we determined the effects of targeted inhibition of PFK1 or FBP1. We first expressed a kinase-deficient variant of the liver isoform (PFKFB1) of 6-phosphofructo-2-kinase/fructose-2,6-bisphosphatase (PFK2-KD), which functions as a constitutively active bisphosphatase and depletes fructose-2,6-P_2_ (14), an activator of PFK1 and inhibitor of FBP1 (41). PFK2-KD caused modest (<20%) lowering of pyruvate + lactate formation (Fig. 7*A*) but raised G6P by ∼3-fold (Fig. 7*B*). We next used the citrate analog aurintricarboxylic acid (ATA), which is a potent inhibitor of PFK1 and antagonizes activation by fructose-2,6-P_2_ (42). ATA caused concentration-dependent lowering of pyruvate and lactate formation and increased G6P by 3-fold also at the lowest concentrations tested (Fig. 7*C*). The lowest [ATA] tested (50 μm) lowered metabolism of both [2-^3^H]glucose and [3-^3^H]glucose by 23 ± 3% and 32 ± 2%, respectively (Fig. 7*D*), with greater (*p* < 0.05) fractional inhibition of [3-^3^H]glucose, consistent with inactivation of PFK1 (42). An inhibitor of FBP1 caused concentration-dependent lowering of G6P but without increasing lactate and pyruvate production and with ATP lowering (Fig. 7*E*). Treatment with amino-oxyacetate (AOA), an inhibitor of the aspartate shuttle, to inhibit transfer of NADH equivalents to mitochondria and thereby increase the cytoplasmic NADH/NAD (43) as shown by the increase in lactate/pyruvate ratio (Fig. 7*F*) had no effect on cell G6P (Fig. 7*G*). This establishes that selective targeting of PFK1 and/or FBP1 but not targeting downstream glycolysis with an increase in cytoplasmic NADH/NAD redox state affects G6P levels.

### Candidate effectors of PFK1-increased P_i_

The 3-fold increase in G6P with the PFK1 inhibitor (42) despite modest inhibition of pyruvate and lactate formation (Fig. 7, *C* and *D*) suggests that allosteric effectors of PFK1 could account for the G6P lowering by metformin with concomitant increased glycolysis (Figs. 2*E*; 5, *C*, *E*, and *F*; 6; and 7*A*). Candidate PFK1 activators include AMP, P_i_, NH_4_^+^, fructose 2,6-P_2_, and fructose 1,6-P_2_, and inhibitors include citrate and G3P (41). Fructose 2,6-P_2_ is lowered by metformin (18) and therefore cannot explain the increased glycolysis. P_i_ is a candidate effector because mitochondrial [P_i_] is severalfold higher than cytoplasmic P_i_, and uptake into mitochondria is by electrogenic transport (44). Accordingly mitochondrial depolarization by low metformin (45) would be expected to increase cytoplasmic P_i_. Total cell P_i_ was increased by high DNP (100 μm) as expected (46) and by high (≥500 μm) metformin (Fig. 7*H*). An increase in cytoplasmic P_i_ with negligible change in total cell P_i_ would be expected at lower metformin or DNP with more modest effects on the mitochondrial proton gradient.

### Roles of G3P and mGPDH activity in modulating cell G6P and G6pc expression

**Figure 8.**
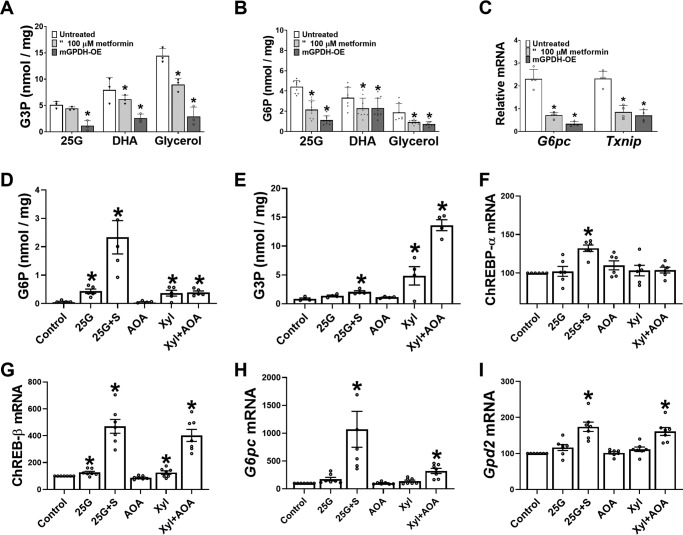
**Overexpression of mGPDH lowers G6P and mimics the metformin repression of *G6pc*.** *A–C*, after cell attachment mouse hepatocytes were either untreated or treated (4 h) with an adenoviral vector (Ad-m-Gpd2 at 4.8 × 10^7^ plaque-forming units/ml) for overexpression of mGPDH (mGPDH-OE). After overnight culture, the hepatocytes were incubated for 2 h in MEM without or with 100 μm metformin (as indicated). *A* and *B*, the medium was then supplemented with 25 mm glucose (*25G*), 5 mm DHA, or 2 mm glycerol, and incubations were for a further 1 h for determination of cell G3P and G6P. *C*, the substrate was 25 mm glucose with additional controls at 5 mm glucose, and incubations were for a further 4 h for RNA extraction and mRNA analysis, which was expressed relative to 5 mm glucose control (1.0). The values are means ± S.E. (*n* = 3–5). *, *p* < 0.05 relative to untreated. *D–I*, after overnight culture, mouse hepatocytes were incubated with either 25 mm glucose (*25G*) without or with S4048 (*25G*+*S*) or with 2 mm xylitol (*Xyl*) or 0.2 mm AOA alone or in combination. Incubations were either for 60 min for determination of cell G6P and G3P (*D* and *E*) or for 4 h (*F–I*) for RNA extraction and analysis of ChREBP-α (*F*), ChREBP-β (*G*), *G6pc* (*H*), and *Gpd2* (*I*) mRNA, which is expressed relative to respective control at 5 mm glucose. The values are means ± S.E. (*n* = 4–5 (*D* and *E*) or *n* = 6–7 (*G–I*)). *, *p* < 0.05 *versus* control.

We showed previously (31) that metformin lowers G3P, a substrate for mGPDH and cGPDH and an inhibitor for PFK-1 (41), in conditions of gluconeogenic flux and proposed a role for the lower G3P in activation of PFK1 and inhibition of gluconeogenesis (31). Liver G3P represents the balance between formation from exogenous glycerol (via glycerokinase) and metabolism by cGPDH and mGPDH. Whereas cGPDH is present at high activity and catalyzes the reversible interconversion of DHAP and G3P, mGPDH is present in low activity in liver and catalyzes the irreversible oxidation of G3P to DHAP (47). We tested the hypothesis that selective lowering of G3P by mGPDH overexpression lowers G6P. Cells overexpressing mGPDH had lower G3P with 25 mm glucose, DHA, and glycerol as substrate (Fig. 8*A*) as expected (31), and they also had markedly lower G6P (Fig. 8*B*). Interestingly, overexpression of mGPDH attenuated the induction of *G6pc* and *Txnip* mRNA by 25 mm glucose, similarly to metformin (Fig. 8*C*).

The *Gpd2* gene encoding mGPDH was identified as a potential target for ChREBP in mouse liver (48). We tested whether *Gpd2* is induced by raised G6P or G3P. Hepatocytes were incubated with either 25 mm glucose with or without S4048 to raise G6P or with the reduced substrate xylitol and without or with AOA to inhibit transfer of NADH equivalents from the cytoplasm and further raise G3P. The highest G6P elevation was with high glucose + S4048 (Fig. 8*D*), and the highest G3P was with xylitol + AOA (Fig. 8*E*). Expression of ChREBP-α was raised by 30% by high glucose + S4048 (Fig. 8*F*), whereas ChREBP-β (Fig. 8*G*), which is a sensitive marker of ChREBP activation (49), was increased by 5- and 4-fold, respectively, by high G6P (25 mm glucose with S4048) and by high G3P (xylitol + AOA) and was also significantly induced by moderate elevation in G6P and G3P with high glucose or xylitol without inhibitors but not with AOA alone. This indicates activation of ChREBP-β by triose-P (G3P), as well as hexose-P (G6P). *G6pc* mRNA was induced to a greater extent by raised G6P compared with raised G3P (Fig. 8*H*), 10-fold *versus* 3-fold (*p* < 0.05), whereas *Gpd2* mRNA was induced similarly (Fig. 8*I*) by raised G3P (61% by xylitol + AOA) and by G6P (74% by 25G + S4048). This shows that the *Gpd2* gene is induced by raised G6P and by G3P (Fig. 8, *D*, *E*, and *I*) and that raised mGPDH activity attenuates the raised G3P and G6P by substrate challenge (Fig. 8, *A* and *B*) and similarly to metformin it attenuates *G6pc* induction by high glucose (Fig. 8*C*). This supports a role for the lower G6P and G3P in the metformin repression of *G6pc*.

### Dinitrophenol and rotenone mimic the G3P lowering by metformin

**Figure 9.**
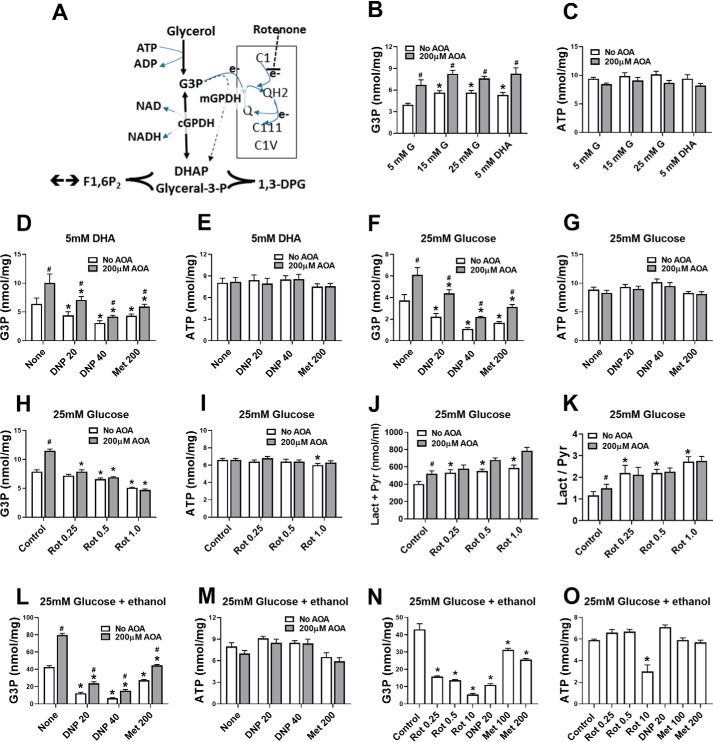
**Effects of amino-oxyacetate and mitochondrial inhibitors on hepatocyte G3P and ATP in mouse hepatocytes.** *A*, cell G3P represents the balance between formation from exogenous glycerol; cGPDH, which catalyzes the reversible NADH/NAD-dependent interconversion of DHAP and G3P; and mGPDH, which catalyzes the irreversible oxidation of G3P to DHAP with transfer of electrons to mitochondrial ubiquinone (*Q*). Rotenone inhibits the transfer of electrons from complex 1 (*C1*) to ubiquinone. *B–O*, hepatocytes were incubated for 1 h in MEM with the substrates indicated without (*open bars*) or with (*filled bars*) 200 μm amino-oxyacetate for analysis of cell G3P and ATP (nmol/mg protein). In *J* and *K*, lactate and pyruvate were determined in the medium of the 60-min incubation. The glucose (*G*) concentration was 5 mm unless otherwise indicated, and ethanol (*L–O*) was 15 mm. Metformin (100 or 200 μm), where indicated (*D–G* and *L–O*), was present in a 2-h preincubation and during the final 1-h incubation. Concentrations of rotenone (*Rot*), DNP, and metformin (*Met*) are shown in μm. The values are means ± S.E. (*n* = 4 (*B*, *C*, *F*, and *G*), *n* = 6 (*D* and *E*), and *n* = 2 (*H–O*) hepatocyte preparations each with triplicate incubations). *, *p* < 0.05 relative to respective control; ^#^, *p* < 0.05 effect of AOA.

We next explored the mechanisms for G3P lowering by metformin. In liver cells in the absence of exogenous glycerol, changes in cell G3P result mainly from changes in the cytoplasmic NADH/NAD redox state via the cGPDH equilibrium (Fig. 9*A*) or from changes in mGPDH activity, which transfers the electrons from G3P oxidation to the mitochondrial ubiquinone pool (Fig. 9*A*). Cell G3P was moderately raised by elevated glucose (15–25 mm) and by 5 mm DHA and was further raised by AOA, whereas ATP was unchanged (Fig. 9, *B* and *C*). The increase by AOA is explained by the raised cytoplasmic NADH/NAD ratio (43) as shown by the raised lactate/pyruvate ratio (Fig. 7*F*), which increases conversion of DHAP to G3P by cGPDH (Fig. 9*A*). The uncoupler DNP, which stimulates mitochondrial pyruvate oxidation by dissipation of the mitochondrial proton gradient, mimicked metformin and lowered G3P, with both DHA (Fig. 9, *D* and *E*) and 25 mm glucose (Fig. 9, *F* and *G*) irrespective of the presence of AOA, with no change in ATP. The DNP effect on G3P is consistent with a more oxidized ubiquinone redox state, by dissipation of the proton gradient. Rotenone (0.25–1 μm), an inhibitor of complex 1, caused concentration-dependent lowering of G3P in the presence of AOA and more modest lowering of G3P at ≥0.5 μm in the absence of AOA (Fig. 9*H*) with mild lowering of ATP at 1 μm rotenone (Fig. 9*I*). In the absence of AOA low rotenone (0.25 μm) raised lactate plus pyruvate production and the lactate/pyruvate ratio (Fig. 9, *J* and *K*), indicating a raised cytoplasmic NADH/NAD. The latter predicts G3P elevation via cGPDH. Accordingly, rotenone has opposite effect on G3P via cGPDH and mGPDH. In the presence of ethanol, which markedly raised cell G3P (Fig. 9, *L–O*), as expected (50), the fractional lowering of G3P by DNP was increased from 40–70% to 70–85% (Fig. 9*L versus* 9*F*), and the lowering of G3P by rotenone was also increased (Fig. 9, *N versus H*). Metformin (100–200 μm) modestly lowered G3P in all conditions tested (Fig. 9, *D*, *F*, *L*, and *N*). Because metformin raises the lactate/pyruvate ratio (Fig. 7*F*), the metformin effect on G3P cannot be explained by the cytoplasmic NADH/NAD redox state and therefore indicates increased mGPDH activity. Cumulatively, G3P lowering by DNP, rotenone, and metformin is best explained by a more oxidized mitochondrial ubiquinone redox state.

## Discussion

We show in this study that metformin lowers G6P in hepatocytes challenged with gluconeogenic precursors or high glucose by increased flux through glycolysis, downstream of G6P and not through effects on glucose phosphorylation or glycogen metabolism. This G6P lowering manifests over a wide range of cell metformin concentrations from the therapeutic range (1–2 nmol/mg cell protein) to 10-fold higher and is not mediated by AMPK-dependent mechanisms. It is a candidate mechanism for the repression of *G6pc* by low metformin in animal models of diabetes (12, 13) and in hepatocytes from AMPK-deficient mice (10).

The induction of *G6pc* (encoding glucose 6-phosphatase) by high glucose is linked to raised metabolites of glucose rather than to glucose itself via activation of the transcription factor ChREBP, which binds to the liver *G6pc* gene promoter (14, 15) and is explained by the adaptive role of glucose 6-phosphatase in maintaining cellular homeostasis of ATP and hexose phosphates (16, 17). We show that *Gpd2* (encoding mGPDH), which is a candidate target gene of ChREBP (48), is induced by raised phosphate esters in conditions of ChREBP-β induction. This indicates an analogous role for mGPDH as for glucose 6-phosphatase (*G6pc*) in cell phosphate ester homeostasis in conditions of glucose excess. We also show that overexpression of mGPDH attenuates the elevation in both hexose phosphate and triose phosphate and mimics the *G6pc* repression by metformin, thus supporting a role for lowering of phosphometabolites in the metformin mechanism on *G6pc*.

The metformin efficacy in G6P lowering manifests in conditions of raised intracellular metabolites, as occurs with high glucose or gluconeogenic precursors, and is further enhanced by the G6P transport inhibitor (S4048), which further enhances G6P elevation (24–26). The finding that the lowering of G6P by metformin was abolished with a glucokinase inhibitor, which lowers G6P is of interest because metformin is known to be ineffective in maturity-onset diabetes of the young linked to inactivating mutations in the *GCK* gene (51). This further supports a role for the G6P lowering in the therapeutic effect of metformin in type 2 diabetes (2).

In the liver, several metabolic pathways can contribute to the G6P lowering by metformin. G6P is generated by glucose phosphorylation, glycogen degradation, and gluconeogenesis and metabolized by glycogen synthesis, glycolysis, pentose pathway, and other minor pathways. We can firmly exclude inhibition of glucose phosphorylation because only very high metformin (>1 mm) inhibited glucose phosphorylation in association with ATP depletion. We also exclude metformin effects on glycogen metabolism (synthesis or degradation) as a cause of the G6P lowering because metformin decreased rather than increased glycogen synthesis and storage, at all concentrations tested, in agreement with previous findings (52). G6P is a key activator of glycogen synthase (37) and inhibitor of glycogen phosphorylase (53). Accordingly, the correlation between G6P lowering and inhibition of glycogen synthesis with increasing metformin points to the decline in G6P as the primary mechanism with attenuation of glycogen synthesis, as secondary to G6P depletion, through partitioning toward glycolysis. The pentose pathway uses G6P as substrate and generates NADPH. Flux through this pathway is determined by the NADP/NADPH ratio (54) and is increased when other pathways for NADPH production (such as NNT, which is driven by the mitochondrial proton gradient) are compromised or when there is increased consumption of NADPH, for example by urea synthesis (34). We considered this pathway as a potential mechanism because metformin raised NADP, and in addition, an NNT inhibitor and ammonium ion, which raise NADP by different mechanisms, both lowered G6P. However, the data on NNT-deficient hepatocytes indicate a mechanism independent of NNT, and furthermore the depletion of G6P by NH_4_^+^ can be explained by allosteric activation of PFK1 (41).

A role for activation of glycolysis at PFK1 by low metformin is supported by various sets of evidence. First, metabolism of [2-^3^H]glucose and [3-^3^H]glucose, which measure respectively, glucose phosphorylation and downstream G6P metabolism by glycolysis and/or pentose pathway showed higher stimulation by metformin of detritiation of [3-^3^H]glucose compared with [2-^3^H]glucose. Second, lactate isotopomers (m2/m1 ratio) from [1,2-^13^C]glucose revealed both a relative increase in glycolysis *versus* pentose pathway and an absolute increase in glycolysis. Third, the citrate analog (ATA) which is a potent inhibitor of PFK1 (42) caused 3-fold elevation in G6P despite modest inhibition of glycolysis, whereas targeting downstream glycolysis with a more reduced cytoplasmic NADH/NAD redox state had negligible effect on G6P. This cumulatively supports a role for allosteric regulation at a proximal site of glycolysis after hexose 6-P formation by targeting of PFK1 and/or FBP1.

FBP1 is inhibited by fructose-2,6-P_2_ and AMP, whereas PFK1 is inhibited by citrate and G3P and activated by fructose 2,6-P_2_, AMP, P_i_, NH_4_^+^, and other effectors (41). Fructose 2,6-P_2_, which has a major role in hepatic regulation of glycolysis by hormones and high glucose, can be excluded from the metformin mechanism on glycolysis and G6P because it is lowered by metformin (18). A somewhat analogous mechanism occurs in hepatocytes during anoxia, which promotes glycolysis with concomitant lowering of fructose 2,6-P_2_ (55) but with raised AMP and lowered citrate, which stimulate and inhibit PFK1, respectively (41, 55). Candidate allosteric effectors to explain the stimulation of glycolysis by metformin include: raised AMP and cytoplasmic P_i_ and lowered citrate and G3P. A role for raised AMP in the inhibition of gluconeogenesis by metformin was recently demonstrated using a knockin mouse model for an AMP-insensitive variant of FBP1 (56). Metformin has been shown to lower citrate levels in a metabolomics study on the Zucker diabetic fatty rat (57) and to lower G3P in hepatocytes incubated with gluconeogenic precursors (31, 58). The lowering of G3P by metformin contrasts with the marked elevation during anoxia (55) and is best explained by mitochondrial depolarization, which favors increased flux through mGPDH.

Cytoplasmic G3P levels are determined by the cGDH equilibrium through changes in the NADH/NAD redox and by the activity of mGPDH, which oxidizes cytoplasmic G3P by transfer of electrons to ubiquinone in the electron transport chain. mGPDH has low affinity for its substrate G3P (47) and thereby a minor role on cell G3P at low substrate when changes in cell G3P would predominantly reflect changes in the cytoplasmic NADH/NAD redox state through the cGDH equilibrium. Here we used high substrate or ethanol to raise G3P. Lowering of G3P by rotenone and metformin in conjunction with an increase in the lactate/pyruvate ratio, which implicates an increase in the G3P/DHAP via the cGDH equilibrium, indicates increased flux through mGPDH. The studies with the uncoupler support a role for mitochondrial depolarization in lowering G3P and G6P and also raising cell P_i_ (an activator of PFK1). Cumulatively this supports multiple allosteric effectors including lower G3P and citrate (57) and raised P_i_ and AMP (56) in the metformin activation of PFK1. It does not exclude a role for the raised AMP causing inhibition of FBP1 as shown recently with a knockin mouse model for an AMP-insensitive FBP1 variant (56).

Two widely studied mechanisms of metformin are the inhibition of complex 1 (19, 59–63) and the activation of AMPK (63, 64). The latter can occur either by a “canonical” pathway downstream of inhibition of complex 1 and consequent mitochondrial depolarization resulting in compromised phosphorylation potential and thereby raised AMP or by a “noncanonical” pathway linked to sensing of fructose 1,6-P_2_ (65, 66). Arguments in support of involvement of complex 1 in activation of AMPK by the canonical pathway have been reviewed (63). The noncanonical pathway occurs in conditions of depletion of fructose 1,6-P_2_, which promotes formation of an AMPK multiprotein complex at the lysosome comprising AXIN, LKB1, Ragulator, v-ATPase, and aldolase functioning as the fructose 1,6-P_2_ sensor (65, 66). We can exclude a role for AMPK activation in the G6P-lowering mechanism because metformin was equally effective in AMPK-KO hepatocytes. However, we cannot exclude involvement of other stress kinases like PKD and MK2 that may be activated through LKB1-independent mechanisms at high metformin (67). In this study, high substrate challenge showed trends of lower AMPK phosphorylation basally and with low metformin (0.2 mm) but enhanced AMPK phosphorylation with high metformin (0.5 mm), indicating that raised hexose phosphates do not antagonize AMPK activation by the canonical pathway.

A key outstanding and contentious issue is whether inhibition of complex 1 is involved the therapeutic effects of metformin (63, 64). Inhibition of the oxygen consumption rate (19, 59) or glucose (pyruvate) oxidation by high metformin are consistent with inhibition of complex 1 and mimicked by rotenone. In this study we found inhibition of glucose oxidation by 0.5 mm but not by 0.2 mm metformin, and this concurs with studies showing inhibition of oxygen consumption at ≥ 0.3 mm metformin (68). Although high metformin (≥0.5 mm) promotes a more reduced mitochondrial NADH/NAD ratio, consistent with complex 1 inhibition (31), low metformin (≤0.2 mm) promotes a more oxidized NADH/NAD ratio and increased β-octanoate oxidation, implicating increased electron transport (31). Nonetheless, glucose (pyruvate) oxidation was unchanged at low metformin (0.2 mm). We cannot exclude a role for targeting of complex 1 by low metformin, whereby metformin causes uncoupling of proton pumping as proposed by Cameron *et al.* (62), and the consequent mitochondrial depolarization favors increased electron transport and increased flux through mGPDH. Depolarization of mitochondria by low metformin has been reported (45). Whether mechanisms independently of complex 1 can explain such a depolarization remains speculative (31). Cumulatively, this study shows that the lowering of G6P in conditions of substrate challenge by metformin is mimicked by mitochondrial depolarization with an uncoupler or by a complex 1 inhibitor and that multiple allosteric effectors of PFK1, including lower G3P and raised P_i_, as well as raised AMP (56) and lower citrate (57), can contribute to the increased disposal of G6P by glycolysis.

## Experimental procedures

### Reagents

A-769662 was from Tocris Biosciences; the AMPK activator C-13 was from the Division of Signal Transduction Therapy, University of Dundee; compound 991 was synthesized by SpiroChem (69); the GKA Ro28-1675 was from Axon MedChem BV (Groningen, The Netherlands); the FBP1 inhibitor 5-chloro-2-[*N*-(2,5-dichlorobenzenesulfonamide)]-benzoxazole (14) was from Calbiochem/Santa Cruz; and S4048 (1-[2-(4-chloro-phenyl)-cyclopropylmethoxy]-3,4-dihydroxy-5-(3-imidazo[4,5-b]pyridin-1-yl-3-phenyl-acryloyloxy)-cyclohexanecarboxylic acid) was a kind gift from Sanofi–Aventis. All other reagents were from Sigma or Tocris Biosciences.

### Hepatocyte isolation and culture

The mice were housed in environmental conditions as outlined in the Home Office Code of Practice. All animal procedures conformed to Home Office Regulations and were approved by the Animal Welfare Ethics Review Board of the Newcastle University Ethics Committee. Hepatocytes were isolated from adult male Wistar rats (Envigo, Bicester, UK) and adult male mice, by collagenase perfusion of the liver (31). Unless otherwise indicated, the mice were of the C57BL/6JOlaHSD strain (Envigo). For the experiments in Fig. 3 on liver AMPK-deficient mice, these were generated by crossing AMPKα1^lox/lox^,α2^lox/lox^ mice (control) against Alfp-Cre (albumin promoter with α-fetoprotein enhancer) to generate AMPKα1^lox/lox^,α2^lox/lox^–Alfp-Cre (AMPK-KO) mice, as previously described (69). For experiments on Nnt-deficient mice, the strain was C57BL/6J (35) from Charles River. For the experiments in Fig. 9, the mice were heterozygous for P446L substitution in the *Gckr* gene (70). The hepatocytes were suspended in minimum essential medium (MEM) containing 5% (v/v) new born calf serum and seeded on gelatin-coated (0.1%) multiwell plates, glass coverslips for immunostaining (18), or glass flasks for ^14^CO_2_ entrapment. After cell attachment the medium was replaced by serum-free MEM containing 5 mm glucose, 10 nm dexamethasone, and 1 nm insulin, and the experiments were started after ∼20 h of culture. For experiments involving enzyme overexpression, the hepatocytes were incubated with adenoviral vectors for 4 h after cell attachment. For overexpression of mouse mGPDH, the adenoviral vector (Ad-m-Gpd2, ADV-279685, 5 × 10^7^ plaque-forming units/ml) was generated by Vector Biolabs (Malvern, PA). The vector for expression of a kinase-deficient bisphosphatase active variant (S32D/T55V) of 6-phosphofructo-2-kinase-fructose-2,6-bisphosphatase, PFKFB1 (denoted by PFK-KD), was described in Ref. 14.

### Hepatocyte incubations

After overnight culture hepatocytes were preincubated for 2 h in MEM containing 5 mm glucose and the concentrations of metformin and AMPK activators or enzyme inhibitors as indicated. The medium was then supplemented with glucose or gluconeogenic substrates and other additions as indicated or replaced by fresh medium with the additions including metformin at the same concentration as for the 2-h preincubation and incubations were continued for 1 h. Where indicated, S4048 (G6P transport inhibitor) was used to raise cell G6P at a concentration of 0.2–2 μm.

Accumulation of [^14^C]metformin was as described in Ref. 18, and metabolism of [U-^14^C]glucose to glycogen and [2-^3^H],[3-^3^H]glucose to ^3^H_2_O were as described in Ref. 71. For [U-^14^C]glucose oxidation, the cells were cultured in glass flasks, and for the final incubation with [U-^14^C]glucose, the flasks contained a 2-ml tube and were sealed with rubber stoppers. The incubation was terminated by injection of HCl (0.2 m final) to the flask, and Hyamine® 10× hydroxide (300 μl; PerkinElmer) was injected into the 2-ml tube to trap the ^14^CO_2_ released by acidification of the medium. For determination of cell metabolites other than P_i_, the medium was aspirated on termination of the incubations, and the hepatocyte monolayers in multiwell pates were snap-frozen in liquid nitrogen and stored at −80 °C until analysis. For determination of cell ATP, G6P and G3P cells were extracted in 2.5% (w/v) sulfosalicylic acid and deproteinized, and ATP was determined by chemiluminescence and G3P and G6P fluorometrically (excitation, 530 nm; emission, 590 nm) as in Ref. 31. For NADP assay, the cells were extracted in 0.6 m HClO_4_, and after deproteinization and neutralization (3 m KOH/1 M K_2_HPO_4_), NADP was assayed fluorometrically (excitation, 340; emission, 450) with yeast glucose 6-phosphate dehydrogenase. For determination of P_i_ on termination of the incubations, the hepatocyte monolayers were rapidly washed two times with 300 mm sucrose, 3 mm HEPES, pH 7.4, and extracted in 0.6 m perchloric acid (4 °C). The extracts were centrifuged (9000 × *g*, 10 min, 4 °C) and assayed immediately as described in Ref. 72. Pyruvate and lactate in the medium were determined by either absorbance (*A*_340 nm_) or fluorometrically (excitation, 340 nm; emission, 450 nm) using lactate dehydrogenase as previously described (31).

### Flux analysis from [1,2-^13^C_2_]glucose

Hepatocytes were incubated in MEM without glutamine, containing 15 mm glucose with [1,2-^13^C_2_]glucose (50%), 2 μm S4048, 0.4 mm α-cyanocinnamate, 500 μm aminooxyacetate, and other additions as indicated for 1 h. The medium was collected at the start and end of the incubation and derivatized for CG-MS analysis of lactate, glucose, and glutamate, as described in Ref. 73. The results of lactate mass isotopologs are expressed as fractional enrichment or m2/m1 ratio (73).

### Immunostaining and immunoblotting

Immunostaining for glucokinase in hepatocyte monolayers on glass coverslips was with a rabbit GK antibody (H-88, sc7908) as in Ref. 74. For immunoblotting, the hepatocytes were extracted in buffer containing 100 mm KCl, 10 mm EDTA, 20 mm K_2_HPO_4_, 0.1% Triton X-100, 0.5 mm phenylmethylsulfonyl fluoride, 0.5 mm benzamidine, 1 mm DTT, 1 μg/ml calyculin A, and 0.1% protease inhibitor mixture. Samples (20 μg of protein) were resolved by SDS-PAGE (90 V for 15 min and 180 V for 45 min) and transferred onto PVDF membrane (Trans Blot SD semidry; 15 V for 45 min). The membranes were probed for AMPKα and phospho-AMPKα–Thr-172 (New England Biolabs catalog nos. 2532 and 2531), acetyl-CoA carboxylase–S79(P) (New England Biolabs catalog no. 3661), and Gapdh (Hytest catalog no. ABIN153387), and protein bands were visualized by enhanced chemiluminescence (Pierce) and exposure to medical film (Agfa Healthcare). Densitometry was imaged by Bio-Rad GS-800 software.

### mRNA analysis

RNA was extracted from the hepatocyte monolayers with TRIzol (Invitrogen), and cDNA was synthesized from 1 μg of RNA using Moloney murine leukemia virus reverse transcriptase (Promega). Sybr-Green based real-time RT-PCR was performed using a Roche-480 Light Cycler in a volume of 10 μl containing 50 ng of reverse transcribed cDNA and 5 ng of forward and reverse primers for rat-*Gck* (forward, GATACCTGGGGAACAGCAAA; reverse, TAGGTGGAGACCCTGCTGAT); rat *G6pc* (forward, CTACCTTGCGGCTCACTTTC; reverse, ATCCAAGTGCGAAACCAAAC); rat *Pklr* (forward, CTGGAACACCTCTGCCTTCTG; reverse, CACAATTTCCACCTCCGACTC); mouse *Nnt* (forward, GGAAGGGTCAGTTGTTGTGG; reverse, CCGGCTTAGTCGTTTCAAAG); mouse *Gapdh* (forward, GACAATGAATACGGCTACAGCA; reverse, GGCCTCTCTTGCTCAGTGTC); mouse *G6pc* (forward, TGGTAGCCCTGTCTTTCTTT; reverse, TCAGTTTCCAGCATTCACAC); mouse *Txnip* (forward, AACATCCCAGATACCCCAGA; reverse, GTGGGGCTCTCTAGTCTGTGA); mouse *Gpd2* (forward, ACTACCTGAGTTCTGACGTTGAAG; reverse, TAACAAGGGGACGGATACCA); mouse ChREBP-α (forward, CGACACTCACCCACCTCTTC; reverse, TTGTTCAGCCGGATCTTGTC); and mouse ChREBP-β (forward, TCTGCAGATCGCGTGGAG; reverse, CTTGTCCCGGCATAGCAAC).

### Statistical analysis

The results are expressed as means ± S.E. for the number of hepatocyte preparations indicated in the legends. Statistical analysis was by Student's *t* test or by analysis of variance.

## Author contributions

T. M., S. S. C., B. E. F., S. M., A. A., N. S. A.-P., M. C., and L. A. formal analysis; T. M., S. S. C., B. E. F., S. M., A. A., N. S. A.-P., C. A., Z. H. A.-O., M. F., B. V., M. C., and L. A. investigation; T. M., S. S. C., B. E. F., S. M., A. A., N. S. A.-P., M. C., and L. A. visualization; T. M., S. M., and L. A. writing-review and editing; S. M. and L. A. conceptualization; C. A., Z. H. A.-O., M. C., and L. A. supervision; M. C. and L. A. funding acquisition; S. S. C., B. E. F., S. M., A. A., M. F., B. V., M. C., and L. A. methodology; L. A. writing-original draft; L. A. project administration; M. F., B. V., M. C., and L. A. resources.

## Footnotes

4 The abbreviations used are:

G6P: glucose 6-phosphate
AMPK: AMP-activated protein kinase
AOA: amino-oxyacetate
ATA: aurintricarboxylic acid
ChREBP: carbohydrate-response element–binding protein
cGPDH: cytoplasmic glycerophosphate dehydrogenase
DHA: dihydroxyacetone
DNP: dinitrophenol
G3P: glycerol 3-phosphate
FBP1: fructose bisphosphatase-1
mGPDH: mitochondrial glycerophosphate dehydrogenase
PFK1: phosphofructokinase-1
NNT: nicotinamide nucleotide transhydrogenase
GKA: glucokinase activator
MEM: minimum essential medium.

## References

[1] Bailey C. J. (2017) Metformin: historical overview. Diabetologia 60, 1566–1576 10.1007/s00125-017-4318-z 28776081

[2] Natali A., and Ferrannini E. (2006) Effects of metformin and thiazolidinediones on suppression of hepatic glucose production and stimulation of glucose uptake in type 2 diabetes: a systematic review. Diabetologia 49, 434–441 10.1007/s00125-006-0141-7 16477438

[3] Rena G., Hardie D. G., and Pearson E. R. (2017) The mechanisms of action of metformin. Diabetologia 60, 1577–1585 10.1007/s00125-017-4342-z 28776086 PMC5552828

[4] Foretz M., Guigas B., Bertrand L., Pollak M., and Viollet B. (2014) Metformin: from mechanisms of action to therapies. Cell Metab. 20, 953–966 10.1016/j.cmet.2014.09.018 25456737

[5] Baur J. A., and Birnbaum M. J. (2014) Control of gluconeogenesis by metformin: does redox trump energy charge? Cell Metab. 20, 197–199 10.1016/j.cmet.2014.07.013 25100057 PMC4154964

[6] Bonora E., Cigolini M., Bosello O., Zancanaro C., Capretti L., Zavaroni I., Coscelli C., and Butturini U. (1984) Lack of effect of intravenous metformin on plasma concentrations of glucose, insulin, C-peptide, glucagon and growth hormone in non-diabetic subjects. Curr. Med. Res. Opin. 9, 47–51 10.1185/03007998409109558 6373159

[7] Sum C. F., Webster J. M., Johnson A. B., Catalano C., Cooper B. G., and Taylor R. (1992) The effect of intravenous metformin on glucose metabolism during hyperglycaemia in type 2 diabetes. Diabet. Med. 9, 61–65 10.1111/j.1464-5491.1992.tb01716.x 1551312

[8] Christensen M. M., Højlund K., Hother-Nielsen O., Stage T. B., Damkier P., Beck-Nielsen H., and Brøsen K. (2015) Endogenous glucose production increases in response to metformin treatment in the glycogen-depleted state in humans: a randomised trial. Diabetologia 58, 2494–2502 10.1007/s00125-015-3733-2 26271344

[9] He L., and Wondisford F. E. (2015) Metformin action: concentrations matter. Cell Metab. 21, 159–162 10.1016/j.cmet.2015.01.003 25651170

[10] Foretz M., Hébrard S., Leclerc J., Zarrinpashneh E., Soty M., Mithieux G., Sakamoto K., Andreelli F., and Viollet B. (2010) Metformin inhibits hepatic gluconeogenesis in mice independently of the LKB1/AMPK pathway via a decrease in hepatic energy state. J. Clin. Invest. 120, 2355–2369 10.1172/JCI40671 20577053 PMC2898585

[11] Dawed A. Y., Ali A., Zhou K., Pearson E. R., and Franks P. W. (2017) Evidence-based prioritisation and enrichment of genes interacting with metformin in type 2 diabetes. Diabetologia 60, 2231–2239 10.1007/s00125-017-4404-2 28842730 PMC6448905

[12] Heishi M., Ichihara J., Teramoto R., Itakura Y., Hayashi K., Ishikawa H., Gomi H., Sakai J., Kanaoka M., Taiji M., and Kimura T. (2006) Global gene expression analysis in liver of obese diabetic db/db mice treated with metformin. Diabetologia 49, 1647–1655 10.1007/s00125-006-0271-y 16752183

[13] Heishi M., Hayashi K., Ichihara J., Ishikawa H., Kawamura T., Kanaoka M., Taiji M., and Kimura T. (2008) Comparison of gene expression changes induced by biguanides in db/db mice liver. J. Toxicol. Sci. 33, 339–347 10.2131/jts.33.339 18670165

[14] Arden C., Tudhope S. J., Petrie J. L., Al-Oanzi Z. H., Cullen K. S., Lange A. J., Towle H. C., and Agius L. (2012) Fructose 2,6-bisphosphate is essential for glucose-regulated gene transcription of glucose-6-phosphatase and other ChREBP target genes in hepatocytes. Biochem. J. 443, 111–123 10.1042/BJ20111280 22214556

[15] Ma L., Robinson L. N., and Towle H. C. (2006) ChREBP*Mlx is the principal mediator of glucose-induced gene expression in the liver. J. Biol. Chem. 281, 28721–28730 10.1074/jbc.M601576200 16885160

[16] Agius L. (2016) Dietary carbohydrate and control of hepatic gene expression: mechanistic links from ATP and phosphate ester homeostasis to the carbohydrate-response element-binding protein. Proc. Nutr. Soc. 75, 10–18 10.1017/S0029665115002451 26264689

[17] Agius L. (2013) High-carbohydrate diets induce hepatic insulin resistance to protect the liver from substrate overload. Biochem. Pharmacol. 85, 306–312 10.1016/j.bcp.2012.09.019 23022226

[18] Al-Oanzi Z. H., Fountana S., Moonira T., Tudhope S. J., Petrie J. L., Alshawi A., Patman G., Arden C., Reeves H. L., and Agius L. (2017) Opposite effects of a glucokinase activator and metformin on glucose-regulated gene expression in hepatocytes. Diabetes Obes. Metab. 19, 1078–1087 10.1111/dom.12910 28206714

[19] Owen M. R., Doran E., and Halestrap A. P. (2000) Evidence that metformin exerts its anti-diabetic effects through inhibition of complex 1 of the mitochondrial respiratory chain. Biochem. J. 348, 607–614 10.1042/0264-6021:3480607 10839993 PMC1221104

[20] Guigas B., Bertrand L., Taleux N., Foretz M., Wiernsperger N., Vertommen D., Andreelli F., Viollet B., and Hue L. (2006) 5-Aminoimidazole-4-carboxamide-1-β-d-ribofuranoside and metformin inhibit hepatic glucose phosphorylation by an AMP-activated protein kinase-independent effect on glucokinase translocation. Diabetes 55, 865–874 10.2337/diabetes.55.04.06.db05-1178 16567505

[21] Fulgencio J. P., Kohl C., Girard J., and Pégorier J. P. (2001) Effect of metformin on fatty acid and glucose metabolism in freshly isolated hepatocytes and on specific gene expression in cultured hepatocytes. Biochem. Pharmacol. 62, 439–446 10.1016/S0006-2952(01)00679-7 11448453

[22] Wilcock C., and Bailey C. J. (1994) Accumulation of metformin by tissues of the normal and diabetic mouse. Xenobiotica 24, 49–57 10.3109/00498259409043220 8165821

[23] Cook D. E., Blair J. B., and Lardy H. A. (1973) Mode of action of hypoglycemic agents: V. Studies with phenethylbiguanide in isolated perfused rat liver. J. Biol. Chem. 248, 5272–5277 4768899

[24] Härndahl L., Schmoll D., Herling A. W., and Agius L. (2006) The role of glucose 6-phosphate in mediating the effects of glucokinase overexpression on hepatic glucose metabolism. FEBS J. 273, 336–346 10.1111/j.1742-4658.2005.05067.x 16403021

[25] Arden C., Petrie J. L., Tudhope S. J., Al-Oanzi Z., Claydon A. J., Beynon R. J., Towle H. C., and Agius L. (2011) Elevated glucose represses liver glucokinase and induces its regulatory protein to safeguard hepatic phosphate homeostasis. Diabetes 60, 3110–3120 10.2337/db11-0061 22013014 PMC3219956

[26] Grefhorst A., Schreurs M., Oosterveer M. H., Cortés V. A., Havinga R., Herling A. W., Reijngoud D. J., Groen A. K., and Kuipers F. (2010) Carbohydrate-response-element-binding protein (ChREBP) and not the liver X receptor α (LXRα) mediates elevated hepatic lipogenic gene expression in a mouse model of glycogen storage disease type 1. Biochem. J. 432, 249–254 10.1042/BJ20101225 20854262

[27] Cool B., Zinker B., Chiou W., Kifle L., Cao N., Perham M., Dickinson R., Adler A., Gagne G., Iyengar R., Zhao G., Marsh K., Kym P., Jung P., Camp H. S., et al (2006) Identification and characterization of a small molecule AMPK activator that treats key components of type 2 diabetes and the metabolic syndrome. Cell Metab. 3, 403–416 10.1016/j.cmet.2006.05.005 16753576

[28] Xiao B., Sanders M. J., Carmena D., Bright N. J., Haire L. F., Underwood E., Patel B. R., Heath R. B., Walker P. A., Hallen S., Giordanetto F., Martin S. R., Carling D., and Gamblin S. J. (2013) Structural basis of AMPK regulation by small molecule activators. Nat. Commun. 4, 3017 10.1038/ncomms4017 24352254 PMC3905731

[29] Hunter R. W., Foretz M., Bultot L., Fullerton M. D., Deak M., Ross F. A., Hawley S. A., Shpiro N., Viollet B., Barron D., Kemp B. E., Steinberg G. R., Hardie D. G., and Sakamoto K. (2014) Mechanism of action of compound-13: an α1-selective small molecule activator of AMPK. Chem. Biol. 21, 866–879 10.1016/j.chembiol.2014.05.014 25036776 PMC4104029

[30] Qiu B. Y., Turner N., Li Y. Y., Gu M., Huang M. W., Wu F., Pang T., Nan F. J., Ye J. M., Li J. Y., and Li J. (2010) High-throughput assay for modulators of mitochondrial membrane potential identifies a novel compound with beneficial effects on db/db mice. Diabetes 59, 256–265 10.2337/db09-0223 19833880 PMC2797930

[31] Alshawi A., and Agius L. (2019) Low metformin causes a more oxidized mitochondrial NADH/NAD redox state in hepatocytes and inhibits gluconeogenesis by a redox-independent mechanism. J. Biol. Chem. 294, 2839–2853 10.1074/jbc.RA118.006670 30591586 PMC6393620

[32] Moyle J., and Mitchell P. (1973) The proton-translocating nicotinamide-adenine dinucleotide (phosphate) transhydrogenase of rat liver mitochondria. Biochem. J. 132, 571–585 4146799 10.1042/bj1320571 PMC1177622

[33] Sies H., Summer K. H., and Bücher T. (1975) A process requiring mitochondrial NADPH: urea formation from ammonia. FEBS Lett. 54, 274–278 10.1016/0014-5793(75)80091-3 236928

[34] Sies H., Akerboom T. P., and Tager J. M. (1977) Mitochondrial and cytosolic NADPH systems and isocitrate dehydrogenase indicator metabolites during ureogensis from ammonia in isolated rat hepatocytes. Eur. J. Biochem. 72, 301–307 10.1111/j.1432-1033.1977.tb11253.x 13998

[35] Kraev A. (2014) Parallel universes of Black Six biology. Biol. Direct. 9, 18 10.1186/1745-6150-9-18 25038798 PMC4108611

[36] Hampson L. J., and Agius L. (2005) Increased potency and efficacy of combined phosphorylase inactivation and glucokinase activation in control of hepatocyte glycogen metabolism. Diabetes 54, 617–623 10.2337/diabetes.54.3.617 15734835

[37] Villar-Palasí C., and Guinovart J. J. (1997) The role of glucose 6-phosphate in the control of glycogen synthase. FASEB J. 11, 544–558 10.1096/fasebj.11.7.9212078 9212078

[38] Katz J., and Wood H. G. (1963) The use of C14O2 yields from glucose-1- and -6-C14 for the evaluation of the pathways of glucose metabolism. J. Biol. Chem. 238, 517–523 13958489

[39] Crawford J. M., and Blum J. J. (1983) Quantitative analysis of flux along the gluconeogenic, glycolytic and pentose phosphate pathways under reducing conditions in hepatocytes isolated from fed rats. Biochem. J. 212, 585–598 10.1042/bj2120585 6411069 PMC1153132

[40] Thomas A. P., and Halestrap A. P. (1981) The rôle of mitochondrial pyruvate transport in the stimulation by glucagon and phenylephrine of gluconeogenesis from l-lactate in isolated rat hepatocytes. Biochem. J. 198, 551–560 10.1042/bj1980551 7326022 PMC1163301

[41] Hers H. G., and Hue L. (1983) Gluconeogenesis and related aspects of glycolysis. Annu. Rev. Biochem. 52, 617–653 10.1146/annurev.bi.52.070183.003153 6311081

[42] McCune S. A., Foe L. G., Kemp R. G., and Jurin R. R. (1989) Aurintricarboxylic acid is a potent inhibitor of phosphofructokinase. Biochem. J. 259, 925–927 10.1042/bj2590925 2525029 PMC1138608

[43] Berry M. N., Gregory R. B., Grivell A. R., Phillips J. W., and Schön A. (1994) The capacity of reducing-equivalent shuttles limits glycolysis during ethanol oxidation. Eur. J. Biochem. 225, 557–564 10.1111/j.1432-1033.1994.00557.x 7957170

[44] Akerboom T. P., Bookelman H., Zuurendonk P. F., van der Meer R., and Tager J. M. (1978) Intramitochondrial and extramitochondrial concentrations of adenine nucleotides and inorganic phosphate in isolated hepatocytes from fasted rats. Eur. J. Biochem. 84, 413–420 10.1111/j.1432-1033.1978.tb12182.x 639797

[45] Dykens J. A., Jamieson J., Marroquin L., Nadanaciva S., Billis P. A., and Will Y. (2008) Biguanide-induced mitochondrial dysfunction yields increased lactate production and cytotoxicity of aerobically-poised HepG2 cells and human hepatocytes *in vitro*. Toxicol. Appl. Pharmacol. 233, 203–210 10.1016/j.taap.2008.08.013 18817800

[46] Vanstapel F., Waebens M., Van Hecke P., Decanniere C., and Stalmans W. (1990) The cytosolic concentration of phosphate determines the maximal rate of glycogenolysis in perfused rat liver. Biochem. J. 266, 207–212 10.1042/bj2660207 2155606 PMC1131116

[47] Mráček T., Drahota Z., and Houštěk J. (2013) The function and the role of the mitochondrial glycerol-3-phosphate dehydrogenase in mammalian tissues. Biochim. Biophys. Acta 1827, 401–410 10.1016/j.bbabio.2012.11.014 23220394

[48] Poungvarin N., Chang B., Imamura M., Chen J., Moolsuwan K., Sae-Lee C., Li W., and Chan L. (2015) Genome-wide analysis of ChREBP binding sites on male mouse liver and white adipose chromatin. Endocrinology 156, 1982–1994 10.1210/en.2014-1666 25751637 PMC4430618

[49] Kim M. S., Krawczyk S. A., Doridot L., Fowler A. J., Wang J. X., Trauger S. A., Noh H. L., Kang H. J., Meissen J. K., Blatnik M., Kim J. K., Lai M., and Herman M. A. (2016) ChREBP regulates fructose-induced glucose production independently of insulin signaling. J. Clin. Invest. 126, 4372–4386 10.1172/JCI81993 27669460 PMC5096918

[50] Van Schaftingen E., Bartrons R., and Hers H. G. (1984) The mechanism by which ethanol decreases the concentration of fructose 2,6-bisphosphate in the liver. Biochem. J. 222, 511–518 10.1042/bj2220511 6089771 PMC1144206

[51] Chakera A. J., Steele A. M., Gloyn A. L., Shepherd M. H., Shields B., Ellard S., and Hattersley A. T. (2015) Recognition and management of individuals with hyperglycemia because of a heterozygous glucokinase mutation. Diabetes Care 38, 1383–1392 10.2337/dc14-2769 26106223

[52] Otto M., Breinholt J., and Westergaard N. (2003) Metformin inhibits glycogen synthesis and gluconeogenesis in cultured rat hepatocytes. Diabetes Obes. Metab. 5, 189–194 10.1046/j.1463-1326.2003.00263.x 12681026

[53] Agius L. (2015) Role of glycogen phosphorylase in liver glycogen metabolism. Mol. Aspects Med. 46, 34–45 10.1016/j.mam.2015.09.002 26519772

[54] Stincone A., Prigione A., Cramer T., Wamelink M. M., Campbell K., Cheung E., Olin-Sandoval V., Grüning N. M., Krüger A., Tauqeer Alam M., Keller M. A., Breitenbach M., Brindle K. M., Rabinowitz J. D., and Ralser M. (2015) The return of metabolism: biochemistry and physiology of the pentose phosphate pathway. Biol. Rev. Camb. Philos. Soc. 90, 927–963 10.1111/brv.12140 25243985 PMC4470864

[55] Hue L. (1982) Role of fructose 2,6-bisphosphate in the stimulation of glycolysis by anoxia in isolated hepatocytes. Biochem. J. 206, 359–365 6216883 10.1042/bj2060359 PMC1158592

[56] Hunter R. W., Hughey C. C., Lantier L., Sundelin E. I., Peggie M., Zeqiraj E., Sicheri F., Jessen N., Wasserman D. H., and Sakamoto K. (2018) Metformin reduces liver glucose production by inhibition of fructose-1–6-bisphosphatase. Nat. Med. 24, 1395–1406 10.1038/s41591-018-0159-7 30150719 PMC6207338

[57] Dong Y., Chen Y. T., Yang Y. X., Shou D., and Li C. Y. (2016) Urinary metabolomic profiling in Zucker diabetic fatty rats with type 2 diabetes mellitus treated with glimepiride, metformin, and their combination. Molecules 21, E1446 27809246 10.3390/molecules21111446 PMC6273299

[58] Argaud D., Roth H., Wiernsperger N., and Leverve X. M. (1993) Metformin decreases gluconeogenesis by enhancing the pyruvate kinase flux in isolated rat hepatocytes. Eur. J. Biochem. 213, 1341–1348 10.1111/j.1432-1033.1993.tb17886.x 8504825

[59] El-Mir M. Y., Nogueira V., Fontaine E., Avéret N., Rigoulet M., and Leverve X. (2000) Dimethylbiguanide inhibits cell respiration via an indirect effect targeted on the respiratory chain complex I. J. Biol. Chem. 275, 223–228 10.1074/jbc.275.1.223 10617608

[60] Bridges H. R., Jones A. J., Pollak M. N., and Hirst J. (2014) Effects of metformin and other biguanides on oxidative phosphorylation in mitochondria. Biochem. J. 462, 475–487 10.1042/BJ20140620 25017630 PMC4148174

[61] Bridges H. R., Sirviö V. A., Agip A. N., and Hirst J. (2016) Molecular features of biguanides required for targeting of mitochondrial respiratory complex I and activation of AMP-kinase. BMC Biol. 14, 65 10.1186/s12915-016-0287-9 27506389 PMC4977651

[62] Cameron A. R., Logie L., Patel K., Erhardt S., Bacon S., Middleton P., Harthill J., Forteath C., Coats J. T., Kerr C., Curry H., Stewart D., Sakamoto K., Repiščák P., Paterson M. J., et al (2018) Metformin selectively targets redox control of complex I energy transduction. Redox Biol. 14, 187–197 10.1016/j.redox.2017.08.018 28942196 PMC5609876

[63] Glossmann H. H., and Lutz O. M. D. (2019) Pharmacology of metformin: an update. Eur. J. Pharmacol. 865, 172782 10.1016/j.ejphar.2019.172782 31705902

[64] Wang Y., An H., Liu T., Qin C., Sesaki H., Guo S., Radovick S., Hussain M., Maheshwari A., Wondisford F. E., O'Rourke B., and He L. (2019) Metformin improves mitochondrial respiratory activity through activation of AMPK. Cell Rep. 29, 1511–1523.e5 10.1016/j.celrep.2019.09.070 31693892 PMC6866677

[65] Zhang C. S., Hawley S. A., Zong Y., Li M., Wang Z., Gray A., Ma T., Cui J., Feng J. W., Zhu M., Wu Y. Q., Li T. Y., Ye Z., Lin S. Y., Yin H., et al (2017) Fructose-1,6-bisphosphate and aldolase mediate glucose sensing by AMPK. Nature 548, 112–116 10.1038/nature23275 28723898 PMC5544942

[66] Li M., Zhang C. S., Zong Y., Feng J. W., Ma T., Hu M., Lin Z., Li X., Xie C., Wu Y., Jiang D., Li Y., Zhang C., Tian X., Wang W., et al (2019) Transient receptor potential V channels are essential for glucose sensing by aldolase and AMPK. Cell Metab. 30, 508–524.e12 10.1016/j.cmet.2019.05.018 31204282 PMC6720459

[67] Stein B. D., Calzolari D., Hellberg K., Hu Y. S., He L., Hung C. M., Toyama E. Q., Ross D. S., Lillemeier B. F., Cantley L. C., Yates J. R. 3rd, and Shaw R. J. (2019) Quantitative *in vivo* proteomics of metformin response in liver reveals AMPK-dependent and -independent signaling networks. Cell Rep. 29, 3331–3348.e7 10.1016/j.celrep.2019.10.117 31801093 PMC6980792

[68] Neal A., Rountree A. M., Philips C. W., Kavanagh T. J., Williams D. P., Newham P., Khalil G., Cook D. L., and Sweet I. R. (2015) Quantification of low-level drug effects using real-time, *in vitro* measurement of oxygen onsumption rate. Toxicol. Sci. 148, 594–602 10.1093/toxsci/kfv208 26396153 PMC4830255

[69] Boudaba N., Marion A., Huet C., Pierre R., Viollet B., and Foretz M. (2018) AMPK re-activation suppresses hepatic steatosis but its downregulation does not promote fatty liver development. EBioMedicine 28, 194–209 10.1016/j.ebiom.2018.01.008 29343420 PMC5835560

[70] Codner G. F., Mianné J., Caulder A., Loeffler J., Fell R., King R., Allan A. J., Mackenzie M., Pike F. J., McCabe C. V., Christou S., Joynson S., Hutchison M., Stewart M. E., Kumar S., et al (2018) Application of long single-stranded DNA donors in genome editing: generation and validation of mouse mutants. BMC Biol. 16, 70 10.1186/s12915-018-0530-7 29925374 PMC6011369

[71] de la Iglesia N., Mukhtar M., Seoane J., Guinovart J. J., and Agius L. (2000) The role of the regulatory protein of glucokinase in the glucose sensory mechanism of the hepatocyte. J. Biol. Chem. 275, 10597–10603 10.1074/jbc.275.14.10597 10744755

[72] Itaya K., and Ui M. (1966) A new micromethod for the colorimetric determination of inorganic phosphate. Clin. Chim. Acta 14, 361–366 10.1016/0009-8981(66)90114-8 5970965

[73] Marin S., Lee W. N., Bassilian S., Lim S., Boros L. G., Centelles J. J., FernAndez-Novell J. M., Guinovart J. J., and Cascante M. (2004) Dynamic profiling of the glucose metabolic network in fasted rat hepatocytes using [1,2–13C2]glucose. Biochem. J. 381, 287–294 10.1042/BJ20031737 15032751 PMC1133787

[74] Payne V. A., Arden C., Wu C., Lange A. J., and Agius L. (2005) Dual role of phosphofructokinase-2/fructose bisphosphatase-2 in regulating the compartmentation and expression of glucokinase in hepatocytes. Diabetes 54, 1949–1957 10.2337/diabetes.54.7.1949 15983194

